# *Larrea divaricata* Cav. aqueous extract and nordihydroguariaretic acid modulate oxidative stress in submandibular glands of diabetic rats: a buccal protective in diabetes

**DOI:** 10.1186/s12906-019-2636-z

**Published:** 2019-08-22

**Authors:** Ignacio Peralta, Carla Marrassini, María Laura Barreiro Arcos, Graciela Cremaschi, María Rosario Alonso, Claudia Anesini

**Affiliations:** 1 Universidad de Buenos Aires, Consejo Nacional de Investigaciones Científicas y Técnicas (CONICET), Instituto de Química y Metabolismo del Fármaco (IQUIMEFA), Buenos Aires, Argentina; 2 Instituto de Investigaciones Biomédicas UCA-CONICET, Buenos Aires, Argentina; 3 Cátedra de Farmacognosia, Facultad de Farmacia y Bioquímica, Universidad de Buenos Aires, Junín 956 2do piso, Buenos Aires, Argentina

**Keywords:** Submandibular gland, Peroxidase, Superoxide dismutase, Streptozotocin, *Larrea divaricata* Cav

## Abstract

**Background:**

Oxidative stress is an imbalance between the levels of reactive oxygen species (ROS), reactive nitrogen species (RNS) and endogenous antioxidants. The aetiology and pathogenesis of several oral diseases are attributed to this process. The antioxidant enzymes secreted in the saliva by submandibular glands maintain oral health through the scavenging of ROS. The objective of this work was to study the capacity of an aqueous extract of *L. divaricata* (AE), and its majority compound, nordihydroguariaretic acid (NDGA), to modulate the pro-oxidant/antioxidant status in submandibular glands in a model of oxidative stress induced by streptozotocin (STZ) in rats.

**Methods:**

To induce oxidative stress with STZ, a group of animals was treated i.p. with 1 X PBS (control group) and other group was injected i.p. once with STZ (60 mg/kg). Ten days after the treatment, blood samples were taken from the tail vain to determine the glucose levels. Animals with glucose values ≥300 mg/ml were selected.

The submandibular glands of control and STZ treated animals were incubated with either the AE (500 μg/ml) or with NDGA (1.5 μg/ml), and the content of malondialdehyde (MDA), protein carbonyl groups, ROS and RNS, and the activity and expression of peroxidase (Px), superoxide dismutase (SOD) and inducible nitric oxide synthase (iNOS) were assayed.

**Results:**

AE decreased the levels of MDA (^##^*P* < 0.01) and protein carbonyl groups (^#^*P* < 0.05), and modulated the levels of ROS such as hydrogen peroxide (H_2_O_2_)(^##^P < 0.01), superoxide anion (O_2_^.-^) (^#^*P* < 0.05) and nitric oxide (NO) (^#^*P* < 0.05) in relation to the modulation of Px and iNOS expression. NDGA was found to be involved in these effects.

**Conclusions:**

The antioxidant activity of the AE in the submandibular glands would allow the maintenance of the antioxidant pool to prevent oral oxidative diseases.

## Background

Oxidative stress is defined as the imbalance between the levels of reactive oxygen species (ROS), reactive nitrogen species and endogenous antioxidants. Any alteration of the homeostasis leads to an increased production of free radicals that cannot be counteracted by detoxifying mechanisms [1]. Free radicals interact with proteins and genes, which leads to tissue damage. Cardiovascular diseases, neuronal degeneration, and cancer are the result of such imbalance. For example, it is known that the hyperglycaemia observed in diabetes mellitus is responsible for the generation of oxidative stress [2]. Oxidative stress is also involved in the aetiology and pathogenesis of several oral diseases, including the development of dental caries and periodontitis [3]. For example, the expression of antioxidant genes has been observed in patients with periodontitis [4]. Moreover, oral pre-cancerous lesions such as lichen planus and leukoplakia and oral squamous cell carcinoma have been found to be associated with oxidative stress and high levels of malondialdehyde (MDA) in serum, which is a marker of oxidative stress [5, 6].

The antioxidant enzymes secreted in the saliva play a major role in the maintenance of oral health through the scavenging of reactive species such as hydrogen peroxide and superoxide anion and nitrogen reactive species, some of which are produced by the oral microflora [7]. The main antioxidant enzymes present in saliva are superoxide dismutase, catalase, and glutathione peroxidase, which are mainly secreted by the salivary glands such as the submandibular ones [8]. Submandibular glands produce approximately 65–70% of saliva, thus being the main source of the antioxidant enzymes of the oral cavity. Although the parotid glands are larger than submandibular glands, they only produce 20% of the saliva secreted. Therefore, any change in the secretory capacity of submandibular glands will lead to the development of oxidative stress-induced oral diseases. It is known that the structure and function of salivary glands is altered in diabetic patients [9]. The amount of microorganisms in the oral cavity is controlled through appropriate oral hygiene, particularly in diabetic patients who are prone to infections. In this sense, mouthwashes can prevent and/or alleviate oral pathologies, acting as deodorant, antiseptic, disinfectant, analgesic, astringent and antioxidant.

It has previously been demonstrated that streptozotocin (STZ), a drug that induces the development of type 1 diabetes mellitus, generates an imbalance between oxidative and antioxidant systems by decreasing the secretion of peroxidase in submandibular glands [10, 11] This is then, a suitable model to test the antioxidant activity of drugs.

Unlike synthetic antioxidants, which are known to cause severe adverse effects, natural antioxidants, when administered locally or systemically, would protect tissues of the oral cavity from the oxidative stress, acting as adjuvants in the treatment of oral diseases of oxidative origin.

*Larrea divaricata* Cav. (Zygophyllaceae) is a bush that grows in South America and is widely distributed in Argentina. This plant is used in folk medicine for its anti-inflammatory properties and is also known to have antitumoral and immunomodulatory activities and antimicrobial properties [12–15]. The antioxidant compound nordihydroguaiaretic acid (NDGA) has already been reported to be present in this plant [16]. It has previously been demonstrated that the aqueous extract of *Larrea divaricata* (AE) stimulates the secretion of peroxidase in normal rat submandibular glands [17]. The effect of the AE on the pro-oxidant/antioxidant homeostasis in salivary glands of normal rats has been studied elsewhere [18]. However, the effect of the AE on the pro-oxidant/antioxidant homeostasis in submandibular glands subjected to the effects of an oxidantive stressor such as STZ remains to be studied.

The aim of this work was to determine the antioxidant activity of the AE prepared with the leaves of *L. divaricata* in a model of oxidative stress induced by STZ in submandibular glands. The levels of ROS, the degree of lipid peroxidation and protein oxidation, and the activity of antioxidant and pro-oxidant enzymes related to the metabolism of H_2_O_2_ and NO were determined in submandibular glands obtained from STZ-treated rats. The participation of the majority compound of the AE, namely NDGA, was also studied. It is hypothesized that the local administration of the AE as either a mouthwash or as a systemic antioxidant could control the damage induced by oxidative stress in soft oral tissues, particularly in diseases that feature oxidative stress such as diabetes.

## Methods

### Plant material

Leaves of *Larrea divaricata* Cav. used in this study were collected in the province of Cordoba, Argentina and identified using morphological, anatomical and histochemical analyses by Dr. Hernán Gerónimo Bach from the Museum of Pharmacobotany, School of Pharmacy and Biochemistry, University of Buenos Aires. One voucher specimen (BAFC no. 38) was deposited at the Museum of Pharmacobotany. The aqueous extract (AE) was prepared from air-dried leaves. Briefly, 750 mg were infused for 20 min with 10 ml of sterilized boiling distilled water and the supernatant was lyophilised [18].

### NDGA quantification by HPLC

The high performance liquid chromatography (HPLC) analysis was performed in a Varian Pro Star instrument equipped with a Rheodyne injection valve (20 μl) and a photodiode array detector set at 260 nm. A reversed-phase Phenomenex-C18 (2) Luna column (250 mm × 4.6 mm and 5 μ pd) was used. Samples were eluted with a gradient of A: water:acetic acid (98:2) and B: methanol:acetic acid (98:2) from 15% B to 40% B in 30 min; 40% B to 75% B in 10 min; 75% B to 85% B in 5 min and 100% B in 5 min. Solution B (100%) was run for 10 min and back to initial conditions. The flow rate was 1.2 ml/min and the separation was done at room temperature (18–25 °C). The optical density was registered in a Varian Star 5.5 detector (USA). Lyophilised aqueous extracts (10 mg/ml) and the pure standard were dissolved in methanol:water (70:30). The water employed to prepare the working solution was of ultrapure quality (Milli-Q). Methanol (J.T. Baker) and acetic acid (Merck, Argentina) were HPLC grade. A pure standard of NDGA (Sigma, USA, lot 19C-0504) was employed [18].

### Total polyphenol determination

The total polyphenols content was determined by spectrophotometry by the Folin-Ciocalteu’s method using gallic acid as standard. The lyophilised extract was weighed and dissolved in distilled water. Briefly, 1.0 ml of the extract was transferred to separate tubes containing 7.0 ml of distilled water, 0.5 ml of Folin–Ciocalteu’s reagent, and 1.5 ml of a 20% sodium carbonate anhydrous solution. Tubes were allowed to stand at room temperature for 60 min and the absorbance at 765 nm was measured in a UV-vis spectrophotometer. The concentration of polyphenols in the samples was derived from a standard curve of gallic acid ranging from 10 to 50 μg/ml (Pearson’s correlation coefficient: *r*^*2*^ = 0.9996) [19].

### Animals

Female albino Wistar rats (*n* = 40) weighing between 150 and 200 g each were used. Animals were acclimatized to laboratory conditions for 7 days before the experiments and housed in groups of five in stainless steel cages and were kept at 22 ± 2 °C in an illumination controlled room (photoperiod: 14 h light and 10 h darkness); they were fed Purina Chow and allowed to have water ad libitum. Twenty four hours before the experiment, animals were fasted and separated in randomized groups: Twenty animals were treated i.p. with 1 X PBS (control group) and twenty animals were injected i.p. once with streptozotocin (STZ) (60 mg/kg). The number of animals was selected taking into account the statistical procedures.

Ten days after the treatment, blood samples were taken from the tail vain to determine the glucose levels. Accu-check Performa test strips (Roche) were employed. For the study, animals with glucose values ≥300 mg/ml were selected. Animals were sacrificed by cervical dislocation and the submandibular glands were dissected. Animals were handled according to Ethics Committee Guidelines from the Faculty of Pharmacy and Biochemistry, University of Buenos Aires that approved the experiments under Exp-FFyB 220,612–1 and the Guide to the Care and Use of Experimental Animals (DHEW Publication, NIH 80–23).

### Submandibular gland preparations

All experiments were performed on submandibular glands removed from normal and STZ-treated female rats euthanised by cervical dislocation. Free connective tissue, fat and lymph nodes were removed under a magnifying glass and the dissected glands were weighed and incubated in Krebs-Henseleit buffer (pH: 7.4) containing 125NaCl; 4.0 mM KCl; 0.5 mM NaH_2_PO_4_; 0.1 mM MgCl_2_; 1.1 mM CaCl_2_ and 5.0 mM glucose; bubbled with 95% O_2_ and 5% CO_2_ at 37 °C.

Upon reaching equilibrium after 10 min, glands were incubated during 40 min in buffer to allow basal secretion. Glands were treated in vitro as follows: from 5 normal animals, five glands were treated with 500 μg/ml of AE using the opposite glands as normal controls. From the other five animals, five glands were treated with 1.5 μg/ml of NDGA, using the opposite glands as normal controls. The same procedure was applied to STZ-treated animals: from five STZ-treated animals, five glands were treated in vitro with 500 μg/ml of AE and the opposite glands were used as controls. From the remaining five animals, five glands were treated with 1.5 μg/ml of NDGA and the opposite glands used as control. The optimum concentrations of AE and NDGA had previously been determined [18].

The activity of peroxidase and superoxide dismutase were determined in the incubation medium and in glands to determine total activity. For determination of enzyme activity in glands, tissues were homogenised in a Sorvall Omni mixer (DuPont Instruments) in Krebs-Henseleit buffer containing 10^− 4^ M phenylmethylsulfonyl fluoride (PMSF, Sigma) and 10^− 3^ M ethylene diamine tetraacetic acid (EDTA). The homogenate was centrifuged at 5000 x g for15 min at 5 °C and the supernatant was used for the determination of the enzymatic activity. To determine the effect of either the AE or that of NDGA, glands were incubated for 40 min in presence of either the AE or NDGA (one gland of each animal was used to determine the basal values) [18]. Proteins were determined in submandibular gland homogenate by the Lowry’s method.

### Determination of the antioxidant activity

Enzymatic antioxidant activities were determined in gland homogenates and in incubation medium from control glands (without any treatment) and from glands treated with either AE or NDGA from normal and STZ-treated animals.

The peroxidase activity was determined according to Herzog and Fahimi (1973) [20]. Briefly, samples were incubated with 775 μl of 5 × 10^− 4^ M 3,3 diaminobenzidine tetrahydrochloride (DAB, Sigma) and 25 μl of H_2_O_2_ (Parafarm R, 30% v/v diluted 1/86 in distilled water). The reaction was initiated by the addition of H_2_O_2_. DAB without H_2_O_2_ was used as blank. The change in absorbance readings was recorded at 30 s intervals for 5 min using a Shimadzu recording spectrophotometer UV-240 (graphic printer PR-1) set at 465 nm, and the ∆ absorbance/min was calculated. The activity of samples was derived from a standard curve displaying a linear relationship between the enzymatic activity and the ∆ absorbance/min. Measurements were performed in duplicate.

The activity of superoxide dismutase was determined by its ability to inhibit the spontaneous oxidation of adrenaline to adrenochrome, which was measured spectrophotometrically at 480 nm. The enzyme activity was calculated taking into account that 1 U of superoxide dismutase inhibits the auto-oxidation of adrenaline by a 50%. Results were expressed as total peroxidase or superoxide dismutase activity U/ml/g gland = activity of homogenised glands + activity determined in incubation medium (mean ± SEM of values from five measurements) [21].

### Determination of hydrogen peroxide (H_2_O_2_) and superoxide anion levels

For the determination of H_2_O_2_, gland homogenates were incubated with 0.56 mM DAB in a buffer containing 140 mM NaCl, 10 mM potassium phosphate, 5.5 mM dextrose; and 0.01 mg/ml type II horseradish peroxidase (Sigma, St. Louis, MO, USA) as described before [22]. Results were expressed as H_2_O_2_ (M) /g gland. A standard curve of known molar concentrations of H_2_O_2_ in buffered DAB was run in each test.

The levels of superoxide anion were determined through the reduction of nitroblue tetrazolium (NBT) (Sigma, CA, USA) to formazan [23]. Briefly, gland homogenates were incubated with 300 μl of NBT during 30 min. The reaction was stopped with 1 N HCl (Tetrahedron, Buenos Aires, Argentina). The formazan generated was extracted with dioxane (Dorwill, Buenos Aires, Argentina) and the absorbance was measured in a microplate reader at 525 nm (Microplate Reader Benchmark. Bio-Rad, CA, USA). Results were expressed as mmol reduced NBT /g gland.

### Total nitrite determination and immunoblot analysis of inducible nitric oxide synthase (iNOS)

Total nitrites were determined employing the Griess reagent [24]. Gland homogenates or serum samples were incubated with the Griess reagent for 20 min in the dark and the absorbance was read at 540 nm. The levels of total nitrites were calculated by interpolation in a standard curve constructed with known concentrations of nitrites.

For the determination of iNOS levels, glands homogenates from control and STZ-treated animals were dissolved in sample buffer (2% SDS, 10% (v/v) glycerol, 62.5 mM Tris· HCl, pH 6.8, 0.2% bromphenol blue and 10 mM 2-mercaptoethanol). Equal amounts of proteins were separated by SDS-PAGE on 10% polyacrylamide gels and transferred to polyvinylidene difluoride (PVDF) membranes. Non-specific binding sites were blocked with blocking buffer (5% non-fat dry milk containing, 0.1% Tween 20 in 100 mM Tris·-HCl and 0.9% NaCl, pH 7.5,) for 2 h. The PVDF membrane was subsequently incubated with an anti-iNOS antibody (Sigma Chemical Co) for 18 h. An anti-actin antibody (Santa Cruz Biotechnology PAIS) was used as loading control. After washing with PBS-Tween, the membrane was incubated for 1 h with a secondary anti-rabbit antibody conjugated to horseradish peroxidase (HRP) (Amersham Biotech PAIS) diluted 1:2000 in PBS-Tween. Immunoreactive bands were visualized using ECL technology (Amersham Pharmacia PAIS). A densitometric analysis was performed with the Image J software (version 5.1, Silk Scientific Corporation PAIS). Densitometry values of iNOS (arbitrary units) in each lane were normalized to the corresponding actin densitometry values [25].

### Determination of lipid peroxidation

Lipid peroxidation levels were assayed by determining the rate of production of thiobarbituric acid-reactive components expressed as malondialdehyde (MDA), according to Ohkawa et al., (1979) with slight modifications [26]. Briefly, 10 μl of gland homogenate or 10 μl of serum were treated with a mixture of 100 μl of 20% TCA and 0.5% thiobarbituric acid (TBA) and 100 μl of butyl hydroxyl toluene (BHT) (4% in ethanol). The reactive mixture was heated at 90 °C for 30 min. The mixture was allowed to cool down and centrifuged for 10 min at 800 x g. The MDA content was determined spectrophotometrically at 532 nm and 600 nm (unspecific absorbance) and the concentration was determined by calculating the difference between the values measured at 532 nm and 600 nm using the molar extinction coefficient (155 mM^− 1^ cm^− 1^). Finally, the content of MDA was expressed in nmol/g gland.

### Determination of glutathione levels

The levels of reduced glutathione (GSH) were determined according to Moron et al., (1979) [27]. Briefly, immediately after obtaining the homogenates, they were precipitated with 0.1 ml of 25% trichloroacetic acid (TCA). Samples were centrifuged and the precipitate was removed. Free sulfhydryl groups were determined in a total volume of 200 μl. One hundred and thirty four μl of 0.6 mM DTNB (5,5′-dithio-bis-(2-nitrobenzoic acid, Sigma) and 56 μl 0.2 mM sodium phosphate buffer (pH 8.0) were added to 10 μl of each supernatant and the absorbance was read at 405 nm in a UV-VIS Systronics spectrophotometer. Glutathione (Sigma) was used as a standard to calculate μmol GSH/g tissue.

### Protein carbonyl group assay

The assay for detection of protein oxidation was based on the generation of carbonyl groups. The generation of carbonyl groups was detected by derivatisation with 2,4-dinitrophenylhydrazine (DNPH), which leads to the formation of a stable 2,4-dinitrophenyl hydrazone product (DNP). This product was measured at 375 nm. The number of carbonyl groups was expressed as nmol/gland [28].

### Detection of peroxidase and superoxide dismutase by western blot

Gland homogenates (40 μg of protein/lane) were size-fractionated by 12% sodium dodecyl sulfate (SDS)-polyacrylamide gel electrophoresis and transferred to a nitrocellulose membrane. Membranes were incubated for 90 min in Tris-buffered saline (TBS, pH 7.5)-3% non-fat milk, washed and incubated overnight with a 1:200 dilution of a rabbit anti-superoxide dismutase antibody or an anti-peroxidase antibody (Santa Cruz Biotechnology Inc., Santa Cruz, California, USA.). Membranes were washed with TBS-0.05%-Tween 20 and incubated with a 1:1000 dilution of a goat anti-rabbit conjugated horseradish peroxidase antiserum (Sigma, CA//California, USA). The immunodetection was performed with the Western Blot Chemiluminescence reagent kit (NEN Life Science, Boston, USA), according to the manufacturer’s directions. Immunoreactive protein bands were analysed with the Corel Photopaint 9.0 software [18].

### Histological studies

For these studies, gland samples from a group of 10 normal control and 10 STZ-treated animals whose glands were treated in vitro with either the AE extract or with NDGA were fixed in a 10% formalin solution at room temperature. Tissues were embedded in paraffin, and 5 μm sections were cut and stained with Hematoxylin-Eosin, and mounted in glass slides for light microscopy and scanning. Specimens were analysed at 200 x in an Aperio CS Scancope.

### Statistical analysis

The level of significance between two or more treatments in comparison with control groups was assessed by an ANOVA followed by the Dunnett’s test. To determine the level of significance between one treatment and a control group, the Student’s *t* test was used. Differences between means were considered significant if *P* < 0.05.

## Results

### HPLC analysis

**Fig. 1:**
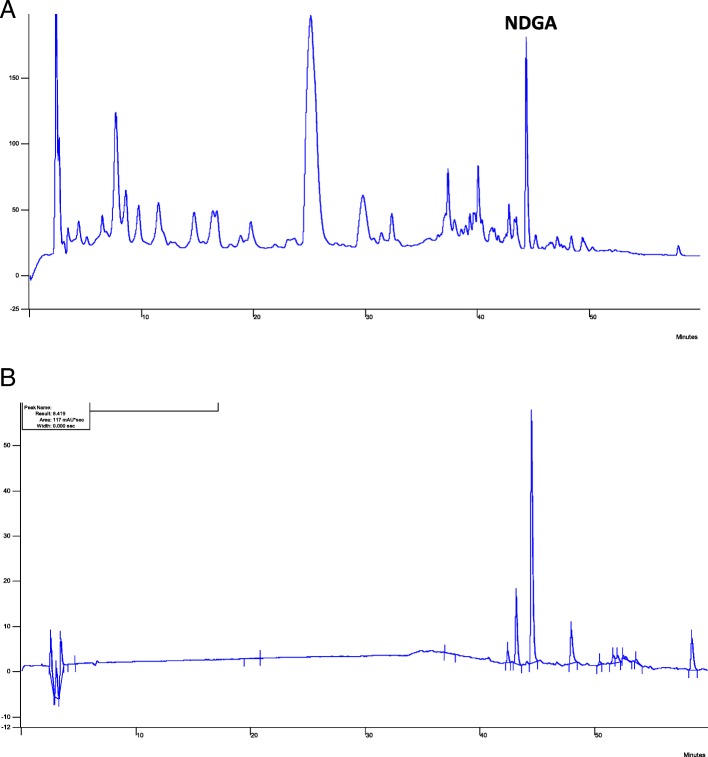
HPLC analysis of the AE obtained from *L. divaricata* Cav. **a**: Chromatographic profile of AE, and (**b**): Chromatographic profile of the NDGA standard

To standardise the AE, the total polyphenol content was first determined. The concentration of polyphenols in the AE was 11.395 ± 0.57 mg GAE/ g extract. The chromatographic profile of the AE showed a peak corresponding to NDGA (0.30 g % w/w) with a retention time at 43.2 min (Fig. 1a and b).

### General biochemical analyses

**Fig. 2:**
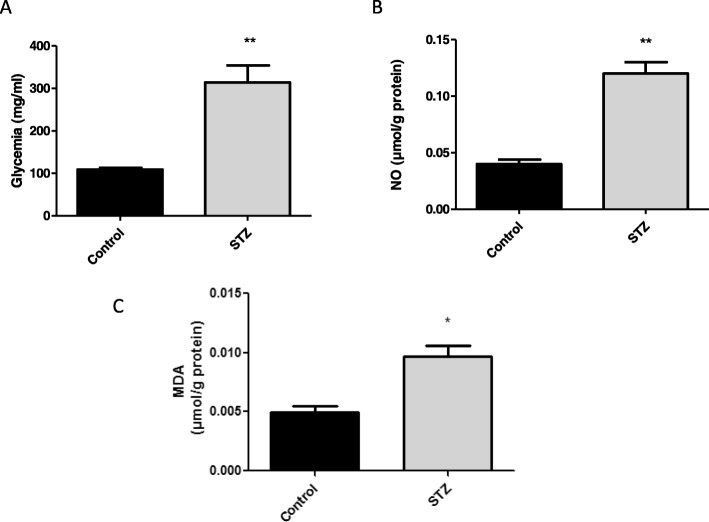
Effects of streptozotocin upon glycemia (**a**), serum NO (**b**) and serum MDA (**c**). Rats were treated with streptozotocin (STZ) and euthanised on day 10 after treatment. Results represent the Mean ± SEM of values from ten controls and ten STZ-treated animals. * *P* < 0.05,** *P* < 0.01 significant differences between control (without any treatment) and STZ-treated animals (Student’s *t* test)

Glycemia, MDA and NO levels were determined in STZ-treated and untreated rats in order to determine if the treatment induced the development of a diabetic status and oxidative stress. STZ-treated rats had higher levels of glycemia (**P < 0.01), MDA (*P < 0.05) and NO (**P < 0.01) than control ones (Fig. 2a, b and c).

### Submandibular gland weight and protein content

**Fig. 3:**
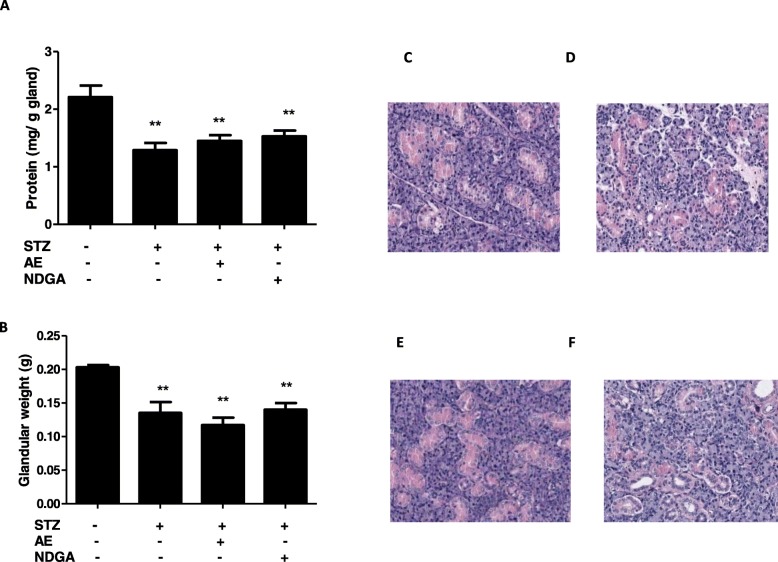
Effect of AE and NDGA on protein content (**a**) weight (**b**), and histology (**c**, **d**, **e**, **f**) in submandibular glands of streptozotocin-treated female rats. Rats were treated with streptozotocin (STZ) and euthanised on day 10 after treatment. **a**: The protein concentration was determined on gland homogenates. **c**, **d**, **e** and **f**: Representative light micrographs (200 X) of rat submandibular glands lobules. Glands were cut, fixed and stained with hematoxylin-eosin. Glands from a control animal (**c**), from a STZ-treated animal (**d**), from a STZ-treated animal + AE (**e**), from a STZ- treated animal + NDGA (**f**). AE: 500 μg/ml, NDGA: 1.5 μg/ml. Results represent Mean ± SEM of values from at least five glands. **P < 0.01 significant differences between submandibular glands from normal control and submandibular glands from STZ- treated animals (Student’s *t* test)

To determine the effect of STZ on submandibular glands, the protein content and weight were determined. Glands obtained from STZ-treated animals had a significant lower weight (**P < 0.01) in comparison with the control (Fig. 3b); the in vitro treatment with either the AE or with NDGA modified neither the weight of normal rats nor that of STZ-treated animals (Fig. 3b). The protein content was lower in STZ-treated glands (**P < 0.01) than in control animals; however, neither AE nor NDGA modified this parameter (Fig. 3a). Moreover, the histologic analysis of the glands revealed that there were alterations in neither the glands acini nor in the ducts of STZ-treated animals, neither in AE or NDGA-treated glands in comparison with controls (Fig. 3c, d, e, f).

### Oxidative parameters in glands

**Fig. 4:**
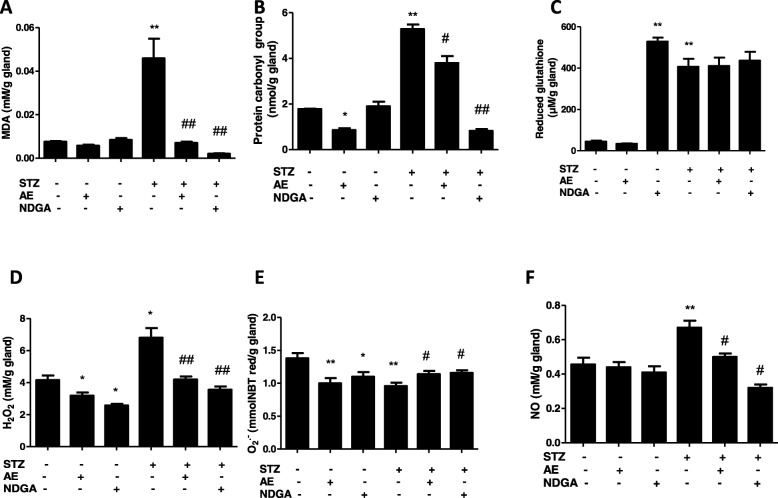
Effect of AE and NDGA on streptozotocin-treated rats on lipid peroxidation (**a**), carbonyl protein groups (**b**), reduced glutathione (**c**), hydrogen peroxide (**d**), superoxide anion (**e**) and NO (**f**) in submandibular glands. Results represent Mean ± SEM of values from at least five glands. * *P* < 0.05; **P < 0.01 significant differences between submandibular glands from normal control and submandibular glands from STZ-treated animals (Student’s *t* test). # *P* < 0.05, ## *P* < 0.01 significant differences between submandibular glands from STZ-treated control animals and STZ-treated glands incubated with either AE or NDGA (ANOVA + Dunnett’s test). AE: 500 μg/ml, NDGA: 1.5 μg/ml

The MDA content was determined to estimate the degree of lipid peroxidation. Figure 4a Shows that glands from STZ-treated animals presented higher levels of MDA (**P < 0.01) than the control group. While the treatment with neither AE nor with NDGA modified the MDA basal levels. These treatments proved to induce a significant decrease in the MDA levels (^##^*P* < 0.01) in STZ-treated animals. In addition, protein carbonyl groups were significantly increased (**P < 0.01) in STZ-treated rats (Fig. 4b). Not only did the treatment with AE decrease (* *P* < 0.05) the number of protein carbonyl groups in basal conditions but also after the STZ treatment (^#^ *P* < 0.05). In contrast, NDGA did not modify the basal levels of protein carbonyl groups, but was capable of decreasing (^##^*P* < 0.01) them in STZ-treated animals. The levels of reduced glutathione were significantly increased (** *P* < 0.01) in glands obtained from STZ-treated animals. NDGA was capable of increasing (** *P* < 0.01) the basal levels of reduced glutathione as well as those in STZ-treated animals. Neither the basal levels nor the levels of reduced glutathione in STZ-treated rats were modified by treatment with the AE (Fig. 4c).

To correlate the levels of lipid peroxidation with the production of reactive oxygen and nitrogen species, the levels of H_2_O_2_, O_2_^.-^ and NO were measured in normal control and STZ-treated rats. The levels of H_2_O_2_ were significantly increased (* *P* < 0.05) in the salivary glands of STZ-treated animals, as compared with control rats (Fig. 4d). Besides, AE and NDGA decreased (*P < 0.05) the levels of H_2_O_2_ under basal conditions and in STZ-treated animals (^##^ *P* < 0.01) (Fig. 4d). In contrast, the levels of O_2_^.-^ were lower (** P < 0.01) in the glands obtained from STZ-treated animals than in controls (Fig. 4e). Although AE and NDGA decreased (** *P* < 0.01 and * *P* < 0.05 respectively) the basal production of O_2_^.-^ they induced an increase (^#^ *P* < 0.05) in STZ-treated animals, though within the range of basal values (Fig. 4e).

Moreover, NO was also significantly increased (** *P* < 0.01) in the salivary glands obtained from STZ-treated animals. The AE and NDGA did not modify the basal production of NO, however, the treatment with both the extract and the pure compound induced a decrease (** *P* < 0.01) in the levels of this mediator in STZ-treated animals (Fig. 4f).

### Effect on antioxidant enzymes

**Fig. 5:**
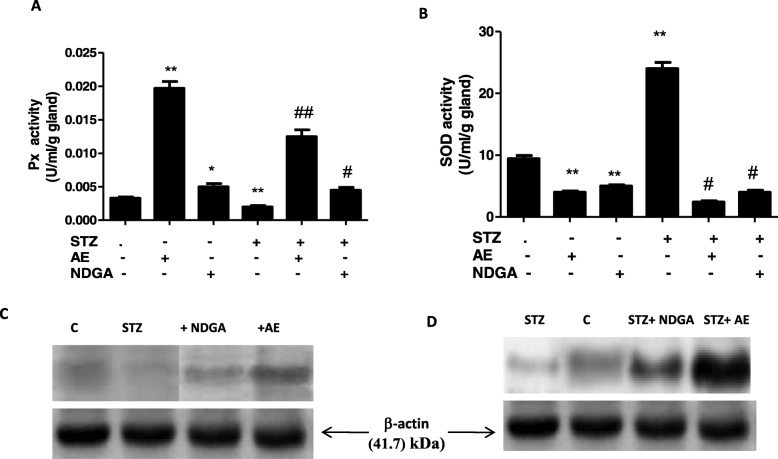
Effect of AE and NDGA on antioxidant enzymes activity (**a** and **b**) and on antioxidant enzymes expression (**c** and **d**) in submandibular glands of STZ- treated rats. Western blots are representative of three experiments. Results represent Mean ± SEM of three determinations made in duplicate. * P < 0.05, ** P < 0.01 significant differences between submandibular glands from normal control and submandibular glands from STZ-treated animals. # P < 0.05, ##P < 0.01 significant differences between glands from STZ and STZ + AE or NDGA-treated glands (ANOVA+ Dunnett’s test). AE: 500 μg/ml, NDGA: 1.5 μg/ml

In order to correlate the levels of reactive oxygen species with the enzymes involved in their metabolism, the total activity of peroxidase and superoxide dismutase were assayed in submandibular glands. The glands from STZ-treated animals presented low peroxidase activity (** *P* < 0.01) and high superoxide dismutase activity (** P < 0.01) (Fig. 5a and b). Under basal conditions and in STZ-treated animals, both AE and NDGA were capable of inducing a significant increase (* *P* < 0.05) and ** *P* < 0.01 respectively) in the peroxidase activity (Fig. 5a), being the increase produced by AE higher than that induced by NDGA. On the contrary, both AE and NDGA decreased the SOD activity under both conditions (** *P* < 0.01 basal condition; ^#^ P < 0.05 STZ – treated condition) (Fig. 5b). To correlate the change in peroxidase and superoxide dismutase activities with a change in enzyme expression, a western blot analysis was performed. The STZ treatment inhibited the synthesis of peroxidase and superoxide dismutase (** *P* < 0.01 and * *P* < 0.05 respectively). However, such inhibition was not observed when glands obtained from STZ-treated rats were treated with either the AE of NDGA (^#^*P* < 0.05 and ^##^ *P* < 0.01 respectively)(Fig. 5c and d and Table 1).

**Table 1:** Relative intensity of immune-reactive protein bands (enzyme/actin)

Glands	Relative intensity of immune-reactive protein bands (enzyme/actin)	
	Px	SOD
Control	1.87 ± 0.1	1.40 ± 0.10
STZ	1 ± 0.1 **	1 ± 0.1*
STZ + NDGA	2 ± 0.2 #	1.47 ± 0.06 #
STZ + AE	3.8 ± 0.4 ##	2.8 ± 0.3 ##

The relative intensity was determined by densitometry analysis of immunoreactive bands with a software. Control: without treatment, *STZ* streptozotocin, *NDGA* nordihydroguaiaretic acid, *AE* aqueous extract of *L. divaricata*, *Px* peroxidase, *SOD* superoxide dismutase. Results represent Mean ± SEM of three determinations made by duplicate. * *P* < 0.05, ** *P* < 0.01significantly differences between submandibulary glands from normal control and submandibulary glands from STZ- treated animals, # *P* < 0.05, ##*P* < 0.01 significantly differences between glands from STZ control and STZ + AE or NDGA treated glands in accordance with ANOVA+ Dunnett’s test. AE: 500 μg/ml, NDGA: 1.5 μg/ml

**Fig. 6:**
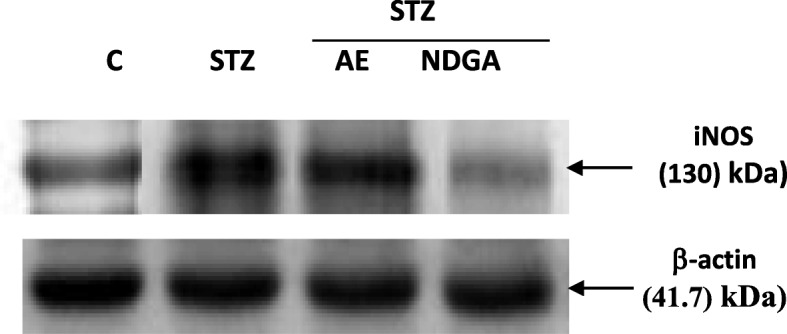
Effect of AE and NDGA on inducible nitric oxide (iNOS) expression in STZ-treated animals. The western blot is representative from three experiments

**Fig. 7:**
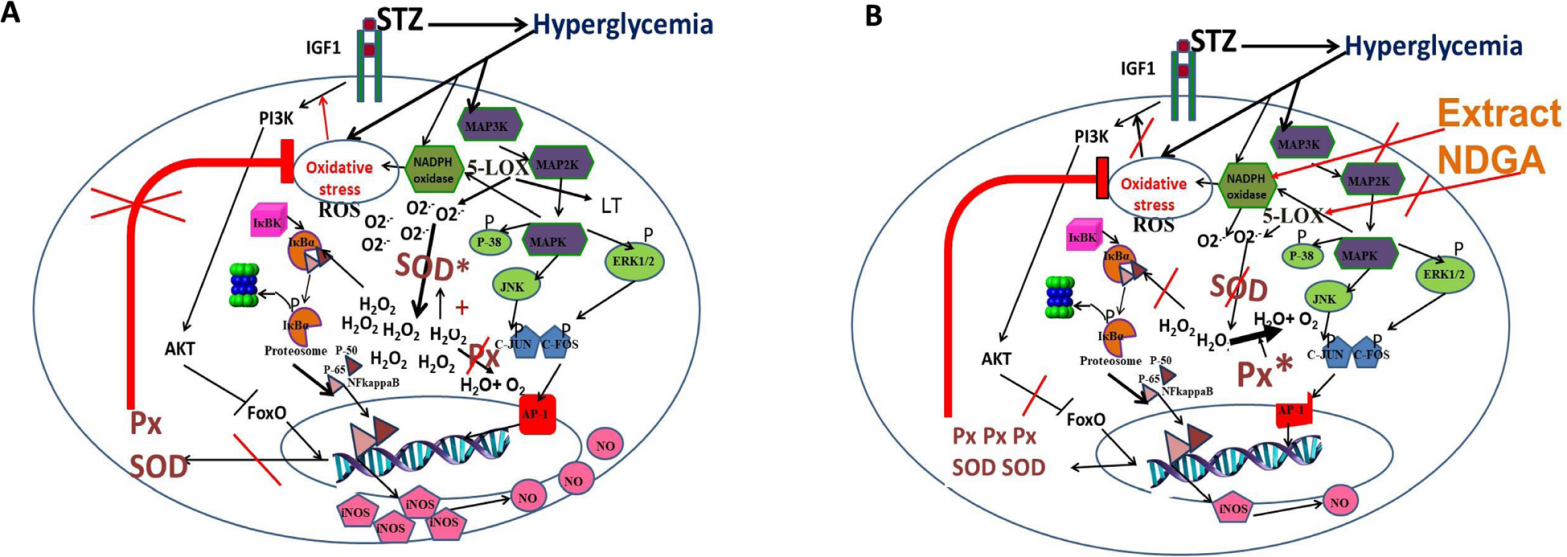
Probable mechanism of the antioxidant action exerted by AE and NDGA on submandibular glands from diabetic rats. **a**- Hyperglycemia induces oxidative stress through MAPK, NADPH oxidase and 5-LOX activation. The ROS generated activate the IGF1R-PI3K-AKT pathway, which inhibites FoxO protein and decreases Px and SOD expression. Besides, Px activity is inhibited, leading to an increase in hydrogen peroxide levels. Hydrogen peroxide, in turn, activates SOD by oxidation and more hydrogen peroxide from superoxide anion is generated. Hydrogen peroxide activates the NFk-B pathway and the up-regulation of iNOS, leading to an increase of NO. **b**- The extract and NDGA inhibit NADPH oxidase and 5-LOX, thus decreasing superoxide anion levels and hydrogen peroxide formation. Low ROS levels activate FoxO protein, which induces the expression of Px and SOD. Moreover, Px is activated, so hydrogen peroxide is transformed into oxygen + water. Low levels of hydrogen peroxide do not activate SOD, therefore, less hydrogen peroxide is formed. NFk-B is not activated therefore, the iNOS expression decreases together with the production of NO.Inhibitory effects are marked in red, while stimulatory effects are marked in black.STZ: streptozotocin, ROS: reactive oxygen species, SOD: superoxide dismutase, Px: peroxidase, 5-LOX: 5 lipooxigenase, iNOS: inducible nitric oxide synthase, FoxO: Forkhead box O transcription factor; AKT: RAC-alpha serine/threonine-protein kinase; IGF1 R: insulin-like factor 1 receptor; PI3K: phosphoinositoside 3 kinase; MAPK: mitogen-activated protein kinase; JNK: c-Jun N terminal kinase; NFκB: nuclear factor kappa-light-chain-enhancer of activated B cells (transcription factor); P-38: P38 mitogen-activated protein kinases; ERK1/2: extracellular signal-regulated kinases

In addition, the expression of iNOS was studied by western blot. The STZ treatment induced (** *P* < 0.01) the expression of iNOS; however, when these glands were treated with AE and NDGA, a decrease (^#^ *P* < 0.05 and ^##^ *P* < 0.01 respectively) in the levels of this enzyme was observed (Fig. 6 and Table 2). The probable mechanism of action of the AE and NDGA is summarized in Fig. 7 as following: A- Hyperglycemia induces oxidative stress through MAPK, NADPH oxidase and 5-LOX activation. The ROS generated activate the IGF1R-PI3K-AKT pathway, which inhibits FoxO protein and decreases Px and SOD expression. Besides, Px activity is inhibited, leading to an increase in hydrogen peroxide levels. Hydrogen peroxide, in turn, activates SOD by oxidation and more hydrogen peroxide from superoxide anion is generated. Hydrogen peroxide activates the NFk-B pathway and the up-regulation of iNOS, leading to an increase of NO. B- The extract and NDGA inhibit NADPH oxidase and 5-LOX, thus decreasing superoxide anion levels and hydrogen peroxide formation. Low ROS levels activate FoxO protein, which induces the expression of Px and SOD. Moreover, Px is activated, so hydrogen peroxide is transformed into oxygen + water. Low levels of hydrogen peroxide do not activate SOD, therefore, less hydrogen peroxide is formed. NFk-B is not activated therefore, the iNOS expression decreases together with the production of NO.

**Table 2:** Relative intensity of immune-reactive protein bands (iNOS/actin)

Glands	Relative intensity of immune-reactive protein bands (iNOS/actin)
Control	0.63 ± 0.06
STZ	1.17 ± 0.08**
STZ+ AE	0.68 ± 0.045#
STZ + NDGA	0.58 ± 0.04 ##

The relative intensity was determined by densitometry analysis of immunoreactive bands with a software. Control: without treatment, *STZ* streptozotocin, *NDGA* nordihydroguaiaretic acid, *AE* aqueous extract of *L. divaricata*, *iNOS* inducible nitric oxide synthase. Results represent the Mean ± SEM of values from tree determinations made by duplicate. * *P* < 0.05, ** *P* < 0.01significantly differences between submandibulary glands from normal control and submandibulary glands from STZ- treated animals, # *P* < 0.05, ##*P* < 0.01 significantly differences between glands from STZ-treated control and STZ-treated + AE or NDGA in accordance with ANOVA+ Dunnett’s test. AE: 500 μg/ml, NDGA: 1.5 μg/ml

## Discussion

In this work the effect of *Larrea divaricata* AE was studied in a model of oxidative stress induced in submandibular glands 10 days after the administration of a single dose of STZ to female rats. The administration of the AE restored the pro-oxidant/antioxidant balance in the glands, thus demonstrating its antioxidant activity.

The NDGA content was determined in the AE by HPLC in order to calculate the concentration of this compound in the AE used in the experiments and to study the participation of NDGA in the overall effects exerted by the extract. The individual effect of NDGA was studied because it is the majority compound of the extract. It is noteworthy that other compounds such as flavonoids have been reported in *L. divaricata;* however, they are in lower quantities than NDGA. The peak observed at RT = 25 min in the HPLC chromatogram is a complex constituted by compounds such as vicenin, rutin and flavonoids measured as quercitrin.

The AE concentration used in the experiments had previously been determined in normal glands [18, 29].

Concentration-response experiments were done with 100, 500 and 1000 μg/ml of AE. The AE was employed at 500 μg/ml, which corresponded to 1.5 μg/ml NDGA. These concentrations produced a maximum effect. The presence of NDGA in this AE has also been described elsewhere [16].

The diabetic status induced by the administration of STZ was corroborated by the high levels of serum glucose, NO and MDA. It is known that the administration of a single dose of 60 mg/kg of streptozotocin triggers an autoimmune process that results in the destruction of the Langerhans’s islet beta cells, thus resulting in the development of clinical diabetes in a short time (2–4 days), and in the induction of oxidative stress [10, 30]. In this context, the submandibular glands are also affected by the treatment with STZ. In fact, a weight decrease of 25%, as compared to control rats was observed in relation to the decrease in protein content. Moreover, signals of oxidative stress were observed in those glands having high levels of MDA, protein carbonylation and reduced glutathione. It is known that MDA reflects the effects of hydroxyl radical damage to cell lipids (lipid peroxidation) under oxidative stress conditions [31].

The increase in the number of carbonyl groups (aldehydes and ketones), which are produced on protein side chains (especially of Pro, Arg, Lys, and Thr), has been observed not only in diabetes but also in Alzheimer’s disease, inflammatory bowel disease, and arthritis [32].

Both the extract and NDGA were capable of reversing the oxidative stress generated in glands by decreasing the levels of MDA and the number of protein carbonyl groups but not those of gluthatione, which remained high, due to an increased synthesis rate. NDGA and the AE inhibited the generation of MDA by 95 and 84%, respectively. These results indicate that the effect of the extract was highly related to the presence of NDGA. On the other hand, the inhibition on protein carbonylation achieved by NDGA was stronger than that achieved by the AE (84% vs. 25%), thus suggesting that the AE has compounds that might counteract the effect exerted by NDGA.

The lipid peroxidation and protein carbonylation induced by the treatment with STZ could be related to the increase of H_2_O_2_ and NO induced by STZ. In fact, the treatment with STZ caused an increase in the levels of H_2_O_2_ and a decrease in O2^.-^ (Fig. 4 d and e). It is known that H_2_O_2_ is generated upon the SOD-catalysed dismutation of O_2_ (Fig. 5b). STZ could be accelerating the transformation of O_2_^.-^ into H_2_O_2_ through the activation of SOD, thus explaining the high levels of H_2_O_2_ and the low levels of O_2_^.-^[33]. On the other hand, STZ deceased the activity of Px and down-regulated its expression (Fig. 5 a and c, Table 1). This finding would also explain the high levels of H_2_O_2_ found in STZ-treated animals.

Moreover, Klotz et al. (2005) have demonstrated that under oxidative stress conditions, ROS such as H_2_O_2_ can activate the insulin-like growth factor 1 receptor (IGF1-R)- phosphatidylinositol 3-kinase (PI3K)-AKT pathway, which inhibits the synthesis of forkhead box (FoxO) protein, which is a transcriptional inducer of antioxidant enzymes [34]. The inhibition of this factor could also explain the down-regulation of Px and SOD exerted by STZ (Fig. 5c and d). It has also been demonstrated that ROS, e.g. H_2_O_2_ activates Cu-Zn-SOD in a concentration-dependent manner by oxidizing the thiol groups of the enzyme during post-transcriptional regulation. The oxidation of its critical thiol group is necessary for the activation of SOD [35]. Taking into account these data, it could be hypothesised that the SOD activation is the result of the increase in the H_2_O_2_ levels induced by STZ (Fig. 5b).

Both the AE and NDGA exerted an antioxidant activity by decreasing not only the levels of H_2_O_2_ but also those of O_2_^.-^. (Fig. 4d and e). The decrease in the H_2_O_2_ levels was achieved by the induction and activation of Px (Fig. 5a and c). Furthermore, the decrease in O_2_^.-^ levels could be related to the NDGA-promoted inhibition of the NADPH oxidase complex, which is involved in the synthesis of O_2_^.-^[36]. However, the latter hypothesis remains to be tested. Beside NADPH-oxidases, cyclooxygenases/lipoxygenases (5-LOX) are generally recognized as the principal physiological sources of O_2_^.-^, which, in turn, dismutates into H_2_O_2_ [37]. It is known that 5-LOX catalyses the production of leukotrienes and ROS from arachidonic acid [38]. The inhibitory capacity of NDGA on LOX would explain the decrease in the levels of O_2_^.-^. The decrease of H_2_O_2_ induced by both the extract and NDGA could, in turn, allow FoxO protein to induce the expression of PX and SOD (Fig. 5c and d Table 1). However, the reduction of H_2_O_2_ levels exerted by the extract and NDGA could not be sufficient to activate Cu-Zn-SOD, whose activity was decreased (Fig. 5b). The same results were obtained with the extract and NDGA in rats not treated with STZ. Both the extract and NDGA were capable of decreasing the levels of H_2_O_2_ and O_2_^.-^ under basal conditions through the activation of Px and the decrease of SOD activity. In line with these results, So Yong Kim et al. (2008) have demonstrated that, as a LOX inhibitor, NDGA scavenges intracellular ROS, inhibits the tumor necrosis factor α (TNF-α)-induced ROS accumulation, blocks the TNF-α-induced NF-κB activation, and inhibits LPS-induced TNF-α production and NF-κB activation in ovalbumin-induced asthma in mice [39].

On the other hand, STZ was capable of increasing the NO levels related to the up-regulation of iNOS. It is known that iNOS generates both O_2_^.-^ and NO, leading to peroxynitrite-mediated cell injury. Therefore iNOS is involved in many diseases associated with inflammation and oxidative stress. It is known that H_2_O_2_ can regulate the expression of iNOS. In fact, it has been demonstrated that H_2_O_2_ stimulates the expression of iNOS in a concentration-response relationship and that H_2_O_2_ can enhance the expression of iNOS induced by cytokines through NFκ-B activation [40, 41]. Therefore, by increasing H_2_O_2_ levels (Fig. 4d), STZ could induce the expression of iNOS (Table 2 and Fig. 6); such effect was counteracted by the extract and NDGA, which decreased H_2_O_2_ levels (Fig. 6 and Table 2). The probable mechanism of action of the extract and NDGA is summarized in Fig. 7.

Even though glands obtained from STZ-treated animals presented a lower weight, as compared to controls, histologic changes were observed in the architecture of neither acini nor ducts, probably due to the short time of the STZ treatment. In a previous study Anderson et al. (1994) only observed a reduction in the acinar cell size after a long treatment (4–6 months) with STZ [42]. Neither degenerative changes in the parenchymal, nor vacuolation, focal loss of salivary architecture or signals of local necrosis or pyknotic nuclei were observed after any treatment (STZ, AE or NDGA), thus demonstrating that neither the extract nor NDGA were cytotoxic at the concentrations assayed.

## Conclusion

The extract of *L. divaricata* was capable of reversing the oxidative stress in the submandibular glands induced by the administration of STZ to female rats. As none of the compounds used in this work had cytotoxic effects on the gland cells, the antioxidant sources remained unaltered, thus being able to prevent the development of oral oxidative diseases.

Although NDGA was demonstrated to be involved in this reversal, the activity of other compounds cannot be ruled out.

The molecular mechanism by which the extract would modulate the oxidative stress would include the modulation of both the activity and the expression of antioxidant and pro-oxidant enzymes, which modulate the levels of oxygen and nitrogen reactive species, preventing MDA formation and protein carbonylation.

Therefore, the extract could be used as a local or systemic preventive agent against oral diseases caused by oxidative stress in diabetic subjects. Further studies are needed to confirm this hypothesis.

## Data Availability

All data are contained and described within the manuscript. The datasets used and/or analyzed during the current study available from the corresponding author on reasonable request.

## Abbreviations

AE: Aqueous extract of *Larrea divaricata*
DAB: Diaminobenzidine tetrahydrochloride
EDTA: Ethylene diamine tetraacetic acid
GSH: Reduced glutathione
H_2_O_2_: Hydrogen peroxide
HRP: Horseradish peroxidase
iNOS: Inducible nitric oxide synthase
MDA: Malondialdehyde
NBT: Nitroblue tetrazolium
NDGA: Nordihydroguariaretic acid
NO: Nitric oxide
O_2_: Superoxide anion
PMSF: Phenylmethylsulfonyl fluoride
PVDF: Polyvinylidene difluoride
Px: Peroxidase
RNS: Reactive nitrogen species
ROS: Reactive oxygen species
SOD: Superoxide dismutase
STZ: Streptozotocin
TBA: Thiobarbituric acid
DTNB: (5,5′-dithio-bis-(2-nitrobenzoic acid trichloroacetic acid (TCA)
DNPH: 2,4-dinitrophenylhydrazine
DNP: 2,4-dinitrophenyl hydrazone
SDS: Sodium dodecyl sulfate
HPLC: High performance liquid chromatography
5-LOX: Lipoxygenases
TNF-α: tumor necrosis factor α
FoxO: Forkhead box
IGF1-R: Insulin-like growth factor 1 receptor
PI3K: Phosphatidylinositol 3-kinase
IGF1 R: Insulin-like factor 1 receptor
PI3K: Phosphoinositoside 3 kinase
MAPK: Mitogen-activated protein kinase
JNK: c-Jun N terminal kinase
NFκB: Nuclear factor kappa-light-chain-enhancer of activated B cells (transcription factor)
P-38: P38 mitogen-activated protein kinases
ERK1/2: Extracellular signal-regulated kinases

## References

[1] Trachootham D, Lu W, Ogasawara MA, Valle NR, Huang P (2008). Redox regulation of cell survival. Antioxid Redox Signal.

[2] FiorentinoTV PA, Zuo P, Folli F (2013). Hyperglycemia-induced oxidative stress and its role in diabetes mellitus related cardiovascular diseases. Curr Pharm De.

[3] Iannitti T, Rottigni V, Palmieri B (2012). Role of free radicals and antioxidant defences in oral cavity-related pathologies. J Oral Pathol Med.

[4] Zeidan-Chulia F, Neves de Oliveir BH, Gursoy M, Kononen E, Fonseca Moreira JC, Gursoy UK, Uitto VJ (2013). MMPREDOX/NO interplay in periodontitis and its inhibition with SaturejahortensisL. Chem Biodivers..

[5] Lopez-Jornet P, Martinez-Canovas A, Pons-Fuste A (2014). Salivary biomarkers of oxidative stress and quality of life in patients with oral lichen planus. Geriatr Gerontol Int.

[6] Metgud R, Bajaj S (2014). Evaluation of salivary and serum lipid peroxidation, and glutathione in oral leukoplakia and oral squamous cell carcinoma. J Oral Sci.

[7] Carlsson J (1987). Peroxidase: an important part of our defence against oxygen toxicity. J Oral Pathol.

[8] Battino M, Ferreiro MS, Gallardo I, Newman HN, Bullon P (2002). The antioxidant capacity of saliva. JClinPeriodontol..

[9] Maekawa ET, Maioral EE, Metidieri HT, Picardi PK, Caldeira EJ (2011). Recovery of INS-R and ER-alpha expression in the salivary glands of diabetic mice submitted to hormone replacement therapy. Arch Oral Biol.

[10] Turner S, Zettler G, Barreiro Arcos ML, Cremaschi G, Davicino R, Anesini C (2011). Effect of streptozotocin on reactive oxygen species and antioxidant enzymes’s secretion in rat submandibulary glands: a direct and an indirect relationship between enzyme activation and expression. Eur J Pharmacol.

[11] Wu KK, Huan Y. Streptozotocin-induced diabetic models in mice and rats. Curr Protoc Pharmacol. 2008; Chapter 5:Unit 5.47. doi: 10.1002/0471141755.ph0547s40. 10.1002/0471141755.ph0547s40 22294227

[12] Del Vitto LA, Petenatti EM (1997). Herbal resource of San Luis (Argentina) first part: native plants. Multequina..

[13] Davicino R, Manuele MG, Turner S, Ferraro G, Anesini C (2010). Antiproliferative activity of Larreadivaricata Cav. On lymphoma cell cine: participation of HydrogenPeroxide in its action. Cancer Investig.

[14] Martino R, Súlsen V, Alonso R, Anesini C (2013). A fraction rich in phenyl propanoids from Larreadivaricata aqueous extract is capable of inducing apoptosis, in relation to H_2_O_2_ modulation, on a murine lymphoma cell line. Leuk Res.

[15] Stege P, Davicino R, Vega A, Casali Y, Correa S, Micalizzi B (2006). Antimicrobial activity of aqueous extracts of Larrea divaricata Cav. (Jarilla) against helicobacter pylori. Phytomedicine..

[16] Davicino R, Alonso R, Anesini C (2011). “In vivo” and “in vitro” activity of Larreadivaricata Cav. On EL-4 cells. Hum ExpToxicol.

[17] Anesini C, Turner S, Borda E, Ferraro G, Coussio J (2004). Effect of LarreadivaricataCav. Extract and nordihydroguaiaretic acid upon peroxidase secretion in rat submandibulary glands. Pharmacol Res.

[18] Peralta I, Martino R, Zettler G, Alonso R, Filip R, Anesini C (2013). Modulator activity of an aqueous extract from L. divaricata Cav. On basal oxidative and anti-oxidative parameters of Normal rat Submandibulary glands. Int J Indigenous Med Plants.

[19] Hosseinzadeh R, Khorsandi K, Hemmaty S (2013). Study of the effect of surfactants on extraction and determination of polyphenolic compounds and antioxidant capacity of fruits extracts. PLoS One.

[20] Herzog V, Fahimi HDA (1973). New sensitive colorimetric assay for peroxidase using 3,3-diaminobenzidine as hydrogen donors. Anal Biochem..

[21] Carrillo MC, Kanai S, Nokubo M, Kitani K (1991). Derprenyl induces activities of both superoxide dismutase and catalase but not glutathione peroxidase in the striatum of young male rats. Life Sci.

[22] Davicino R, Manuele MG, Ferraro G, Micalizzi B, Anesini C (2009). Modulatory effect of hydrogen peroxide on tumoral lymphocytes proliferation. Immunopharmacol Immunotoxicol.

[23] Schopf RE, Mattar J, Meyenburg W, Scheiner O, Hammann KP, Lemmei EM (1984). Measurement of the respiratory burst in human monocytes and polymorphonuclear leukocytes by nitro blue tetrazolium reduction and chemiluminescence. J Immunol Methods.

[24] Becherel PA, Chosidow O, LeGoff L, Frances C, Debre P, Mossalayi MD, Arock M (1997). Inducible nitric oxide synthase and proinflammatory cytokine expression by human keratinocytes during acute urticaria. Mol Med.

[25] Gorelik G, Barreiro Arcos ML, Klecha AJ, Cremaschi GA (2002). Differential expression of protein kinase C isoenzymes related to high nitric oxide synthase activity in a T lymphoma cell line. Biochim Biophys Acta..

[26] Ohkawa H, Ohishi N, Yagi K (1979). Assay for lipid peroxides in animal tissues by thiobarbituric acid reaction. Anal Biochem..

[27] Moron MA, Depierre JW, Mannervick B (1979). Levels of glutathione, glutathione reductase and glutathione S-transferase activities in rat lung and liver. Biochim Biophys Acta.

[28] Levine RL, Garland D, Oliver CN, Amici A, Climent I, Lenz A, Ahn BW, Shaltiel S, Stadtman ER (1990). Determination of carbonyl content in oxidatively modified proteins. Methods Enzymol.

[29] Peralta I, Martino R, Davicino R, Gorzalczany S, Alonso R, Anesini. Systemic and local toxicity assay of an aqueous extract of *Larrea divaricata* Cav.: Role of NDGA IJSR 2015; 6(7):2790–2798.

[30] Weiss RB (1982). Streptozotocin: a review of its pharmacology, efficacy and toxicity. CancerTreat Rep.

[31] Babu PVA, Sabitha KE, Shyamaladevi CS (2006). Therapeutic effect of green tea extract on oxidative stress in aorta and heart of streptozotocin diabetic rats. ChemBiol Interact.

[32] Halliwell B, Gutteridge J (1999). Free radicals in biology and medicine.

[33] Halliwell B, Gutteridge JM (1990). Role of free radicals and catalytic metal ions in human disease: an overview. Methods Enzymol.

[34] Klotz LO, Sánchez-Ramos C, Prieto-Arroyo I, Pavel U, Steinbrenner H, Monsalve M (2015). Redox regulation of FoxOtranscriptionfactors. Redox Biol.

[35] Iñarrea P, Moini H, Rettori D, Han D, Martinez J, Garcia I, Fernandez-Vizarra E, Iturralde M, Cadenas E (2005). RedoxactivationofmitochondrialintermembranespaceCu,Zn-superoxidedismutase. Biochem J.

[36] Paracatu LC, de Faria CM, Zeraik ML, Quinello C, Rennó C, Palmeira P, da Fonseca LM, Ximenes VF (2015). Hydrophobicity and antioxidant activity acting together for the beneficial health properties of nordihydroguaiaretic acid. Food Funct.

[37] Rhee SG, Chang TS, Bae YS, Lee SR, Kang SW (2003). Cellular regulation by hydrogen peroxide. J Am Soc Nephrol.

[38] Harrison KA, Murphy RC (1995). Isoleukotrienes are biologically active free radical products of lipid peroxidation. J Biol Chem.

[39] Kim SY, Kim T-B, Moon K-a, Kim TJ, Shin D, Cho YS, Moon H-B, Lee K-Y (2008). Regulation of pro-inflammatory responses by lipoxygenases via intracellular reactive oxygen species in vitro and in vivo. Exp Mol Med.

[40] Ebrahimi M, Sadeghizadeh M, Noori-Daloi MR (2002). Expression of inducible nitric oxide synthase gene (iNOS) stimulated by hydrogen peroxide in human endothelial cells. Med J Islam Repub Iran.

[41] Ahn JH, Song JS (1999). The effect of hydrogen peroxide on inducible nitric oxide synthase expression in murine macrophage RAW264.7 cells. Tuberc Respir Dis.

[42] Anderson LC, Suleiman AH, Garret JR (1994). Morphological effects of diabetes on the granular ducts and acini of the rat submandibular gland. Microsc Res Tech.

